# Unrestricted kinematic alignment total knee arthroplasty provides favourable five‐year outcomes regardless of varus severity in Japanese patients

**DOI:** 10.1002/jeo2.70802

**Published:** 2026-06-15

**Authors:** Shuji Toyono, Shigenobu Fukushima, Takao Yamamoto, Takashi Ito, Daizo Saito, Taku Nakajima, Yoshihiro Wanezaki

**Affiliations:** ^1^ Department of Orthopaedic Surgery, Joint Replacement Center Saiseikai Yamagata Saisei Hospital Yamagata Japan; ^2^ Department of Orthopaedic Surgery Yamagata Prefectural Shinjo Hospital Shinjo Japan; ^3^ Department of Orthopaedic Surgery, Faculty of Medicine Yamagata University Yamagata Japan; ^4^ Department of Orthopaedic Surgery Okitama Public General Hospital Yamagata Japan

**Keywords:** Japanese patients, kinematic alignment, patient‐reported outcome measures, total knee arthroplasty, varus osteoarthritis

## Abstract

**Purpose:**

To evaluate 5‐year patient‐reported outcomes and radiographic alignment in Japanese patients undergoing unrestricted kinematic alignment total knee arthroplasty (urKA‐TKA), and to assess whether pre‐operative coronal alignment severity influences mid‐term outcomes.

**Methods:**

This retrospective cohort study included 184 consecutive knees treated with urKA‐TKA using a medial pivot implant (GMK Sphere) between June 2019 and November 2020. Five‐year patient‐reported outcome measures included the Forgotten Joint Score (FJS) and the 2011 Knee Society Score (KSS). Radiographic parameters, including the hip–knee–ankle angle (HKA), mechanical lateral distal femoral angle and medial proximal tibial angle, were assessed pre‐operatively and at final follow‐up. Knees were stratified into four groups based on pre‐operative HKA (0°–5°, 5°–10°, 10°–15° and ≥15°), and outcomes were compared among groups.

**Results:**

Of the 184 knees included, 100 completed 5‐year follow‐up, with complete KSS data available for 90 knees and FJS data for 79 knees. The mean age at surgery was 76 ± 8 years. Mean HKA changed from 11 ± 5° pre‐operatively to 3° ± 4° post‐operatively. At 5 years, the mean FJS was 64 ± 25 and the mean total KSS was 127 ± 29. No significant differences were observed among the four alignment groups in total KSS, KSS satisfaction or functional activity scores. FJS values tended to be higher in knees with severe pre‐operative varus (HKA ≥ 15°); however, this difference did not reach statistical significance across the four groups.

**Conclusions:**

UrKA‐TKA provided excellent 5‐year outcomes, with no revisions observed during the 5‐year follow‐up in Japanese patients with varus osteoarthritis. Pre‐operative coronal alignment severity, including severe varus deformity, did not adversely affect mid‐term clinical outcomes, supporting the safe application of urKA across a wide spectrum of varus alignment.

**Level of Evidence:**

Level III.

**Abbreviations**
BMI: body mass index
CR: cruciate‐retaining
CS: cruciate‐substituting
FJS: Forgotten Joint Score
HKA: hip–knee–ankle angle
JLCA: joint line convergence angle
KA: kinematic alignment
KA‐TKA: kinematic alignment total knee arthroplasty
KSS: Knee Society Score
MA: mechanical alignment
MA‐TKA: mechanical alignment total knee arthroplasty
mLDFA: mechanical lateral distal femoral angle
MPTA: medial proximal tibial angle
PROMs: patient‐reported outcome measures
TKA: total knee arthroplasty
urKA: unrestricted kinematic alignment
urKA‐TKA: unrestricted kinematic alignment total knee arthroplasty

## INTRODUCTION

Kinematic alignment (KA) was introduced to restore each patient′s pre‐arthritic joint line and ligamentous envelope [18, 19]. Comparative studies have reported faster recovery, improved functional outcomes, and higher satisfaction with KA compared with mechanical alignment (MA) [2, 14]. Furthermore, mid‐ to long‐term follow‐up studies have demonstrated that respecting the native joint line does not compromise implant durability [11, 28]. Unrestricted KA (urKA), which avoids predefined coronal alignment boundaries, has therefore gained attention as a more individualised and physiologic alignment strategy.

These considerations may be particularly important for East Asian populations, where constitutional varus morphology is more prevalent [27, 30]. Previous studies from Japanese cohorts have demonstrated favourable short‐term clinical outcomes following kinematic alignment total knee arthroplasty (KA‐TKA), supporting the feasibility of KA in this population [29]. In Japanese patients, such native morphology—characterised by a naturally varus lower limb alignment—may influence the suitability and performance of different alignment strategies in TKA. Severe varus deformity also remains a challenge in MA‐TKA [20], and whether urKA can reliably accommodate such anatomy without compromising outcomes is not fully understood.

The purpose of this study was to evaluate 5‐year clinical outcomes, patient‐reported outcome measures (PROMs), radiographic alignment, and survivorship in Japanese patients undergoing urKA‐TKA. A secondary objective was to compare outcomes across increasing levels of pre‐operative coronal varus severity, including knees with severe varus deformity (HKA ≥ 15°). We hypothesised that urKA would yield excellent mid‐term outcomes and that even knees with pronounced varus deformity would achieve functional results comparable to those with milder deformity.

## MATERIALS AND METHODS

### Study design and patient selection

This retrospective cohort study included consecutive patients who underwent urKA‐TKA at our institution between June 2019 and November 2020. A total of 212 primary TKA procedures were performed during the study period. Knees were excluded if they demonstrated valgus alignment, rheumatoid arthritis, prior high tibial osteotomy, ipsilateral total hip arthroplasty or extra‐articular deformities affecting alignment analysis. After applying these criteria, 184 knees were eligible for radiographic evaluation and included in the baseline cohort.

For the 5‐year clinical analysis, knees were excluded if the patient died during follow‐up, developed unrelated medical conditions that prevented evaluation, voluntarily discontinued follow‐up or were lost to follow‐up.

All TKAs were performed by a team of four board‐certified orthopaedic surgeons, all specialists in knee arthroplasty.

### Surgical technique

All procedures were performed using a mid‐vastus approach under the urKA‐TKA philosophy, as previously described [3, 10]. The femur was resected using a calipered technique to match the thickness of the femoral component, with a 2‐mm adjustment for cartilage wear [10]. The tibia was resected using a manual traction technique, in which the lower leg was drawn distally and the tibial cut was aligned parallel to the distal femoral resection surface, thereby respecting the native soft‐tissue envelope [3]. Medial and lateral gap balance was determined using spacer blocks, assessing the pivot motion to ensure balance in extension. The balance between the medial and lateral compartments was adjusted by performing an additional tibial resection, without using soft tissue release. No restrictions were placed on the tibial bone cutting angle.

Medial pivot implants (GMK Sphere CR or CS; Medacta, Switzerland) were used in all cases. Component fixation was cemented. Patellar resurfacing was performed selectively based on intraoperative findings, including cartilage wear, patellar tracking, and surgeon preference.

### Radiographic evaluation

Long‐leg, weight‐bearing radiographs were obtained pre‐operatively and at the most recent follow‐up. Radiographic measurements included the hip–knee–ankle angle (HKA), representing overall coronal limb alignment; the mechanical lateral distal femoral angle (mLDFA); and the medial proximal tibial angle (MPTA). All angles were defined and measured according to established methods. Intra‐ and interobserver reliability for radiographic measurements was assessed using intraclass correlation coefficients, which ranged from 0.71 to 0.99.

Radiolucent lines were assessed on standardised anteroposterior and lateral radiographs. Tibial radiolucencies were evaluated by zone (Zones 1–4), and progression was defined as enlargement or appearance of new radiolucent lines on serial radiographs [7].

Based on pre‐operative coronal alignment measured by the HKA, knees were stratified into four groups: 0° ≤ HKA ≤ 5°, 5° < HKA < 10°, 10° ≤ HKA < 15° and HKA ≥ 15° [20]. These groups were used for comparison of clinical and radiographic outcomes at 5 years.

### Patient‐reported outcome measures (PROMs)

PROMs were collected pre‐operatively and at 5‐year follow‐up. These included the 2011 Knee Society Score (KSS), comprising the Symptoms, Satisfaction, and Functional Activities domains [22], and the Forgotten Joint Score (FJS), which assesses joint awareness during daily activities [1]. Only patients who completed the full set of questionnaires were included in the PROM analysis.

### Statistical analysis

Continuous variables are presented as mean ± standard deviation. Comparisons among the four pre‐operative alignment groups were performed using the Kruskal–Wallis test. Statistical significance was set at *p* < 0.05. All analyses were performed using GraphPad Prism (version 9.4.1; GraphPad Software, San Diego, CA).

## RESULTS

### Patient cohort

A total of 184 knees met the inclusion criteria and were included in the radiographic analysis. Among these, 100 knees completed 5‐year follow‐up, with complete 5‐year KSS data available for 90 knees; FJS data were available for 79 knees. During the follow‐up period, five patients died of causes unrelated to TKA, eight were classified as medical dropouts, 42 were lost to follow‐up, and 29 discontinued follow‐up at their own request.

Baseline demographic and clinical characteristics according to pre‐operative coronal alignment are summarised in Table 1.

**Table 1:** Baseline patient characteristics according to pre‐operative coronal alignment.

Characteristic	0 ≤ HKA ≤ 5	5 < HKA < 10	10 ≤ HKA < 15	HKA ≥ 15	*p*‐value
Number of knees, *n*	25	56	59	44	—
Age, years	72 ± 11	74 ± 8	78 ± 7	78 ± 7	0.0192
BMI, kg/m^2^	27 ± 5	26 ± 4	26 ± 4	25 ± 4	0.1591
Knee extension, °	−8 ± 6	−7 ± 7	−7 ± 7	−8 ± 7	0.3757
Knee flexion, °	129 ± 15	130 ± 16	131 ± 14	120 ± 17	0.0625
HKA, °	3 ± 2	7 ± 1	12 ± 2	18 ± 2	<0.0001
mLDFA, °	86 ± 2	88 ± 2	90 ± 2	91 ± 2	<0.0001
MPTA, °	87 ± 2	85 ± 2	83 ± 2	81 ± 3	<0.0001

Abbreviations: BMI, body mass index; HKA, hip–knee–ankle angle; mLDFA, mechanical lateral distal femoral angle; MPTA, medial proximal tibial angle.

### Overall 5‐year outcomes

Overall 5‐year clinical and radiographic outcomes of urKA‐TKA are presented in Table 2.

**Table 2:** Overall 5‐year outcomes of unrestricted kinematic alignment total knee arthroplasty.

Outcome	*n*	Pre‐operative	Post‐operative (5 years)
Clinical outcomes			
Forgotten Joint Score (FJS)	79	–	64 ± 25
Knee Society Score (KSS), total	90	80 ± 21	127 ± 29
KSS satisfaction	90	13 ± 7	27 ± 9
KSS functional activities	90	44 ± 14	67 ± 20
Radiographic outcomes			
Hip–knee–ankle angle (HKA), °	184	11 ± 5	3 ± 4
Mechanical lateral distal femoral angle (mLDFA), °	184	89 ± 3	88 ± 3
Medial proximal tibial angle (MPTA), °	184	83 ± 3	86 ± 3
Range of motion			
Knee extension, °	184	−8 ± 7	−2 ± 4
Knee flexion, °	184	128 ± 16	130 ± 15

At 5 years post‐operatively, the mean total KSS was 127 ± 29 points. Subscale scores demonstrated favourable outcomes, with a mean satisfaction score of 27 ± 9 and a mean functional activity score of 67 ± 20. The mean FJS was 64 ± 25, indicating favourable joint awareness at mid‐term follow‐up.

Radiographically, the mean HKA changed from 11 ± 5° pre‐operatively to 3 ± 4° post‐operatively. Mean mLDFA and MPTA were maintained close to native alignment values. Knee range of motion showed a modest improvement, with extension improving from −8 ± 7° to −2 ± 4° and flexion from 128 ± 16° to 130 ± 15°.

### Five‐year clinical outcomes according to pre‐operative coronal alignment

Five‐year clinical outcomes stratified by pre‐operative coronal alignment are summarised in Table 3.

**Table 3:** Five‐year clinical outcomes according to pre‐operative coronal alignment.

Outcome	0 ≤ HKA ≤ 5 (*n* = 11)	5 < HKA < 10 (*n* = 33)	10 ≤ HKA < 15 (*n* = 30)	HKA ≥ 15 (*n* = 16)	*p*‐value
KSS total (0–180)	127 ± 31	127 ± 29	125 ± 33	136 ± 27	0.691
KSS satisfaction (0–40)	27 ± 6	27 ± 10	27 ± 11	27 ± 8	0.979
KSS functional activities (0–100)	68 ± 19	66 ± 19	64 ± 21	72 ± 19	0.646

Abbreviations: HKA, hip–knee–ankle angle; KSS, Knee Society Score.

No significant differences were observed among the four alignment groups in total KSS, KSS satisfaction or KSS functional activity scores at 5 years.

**Figure 1:**
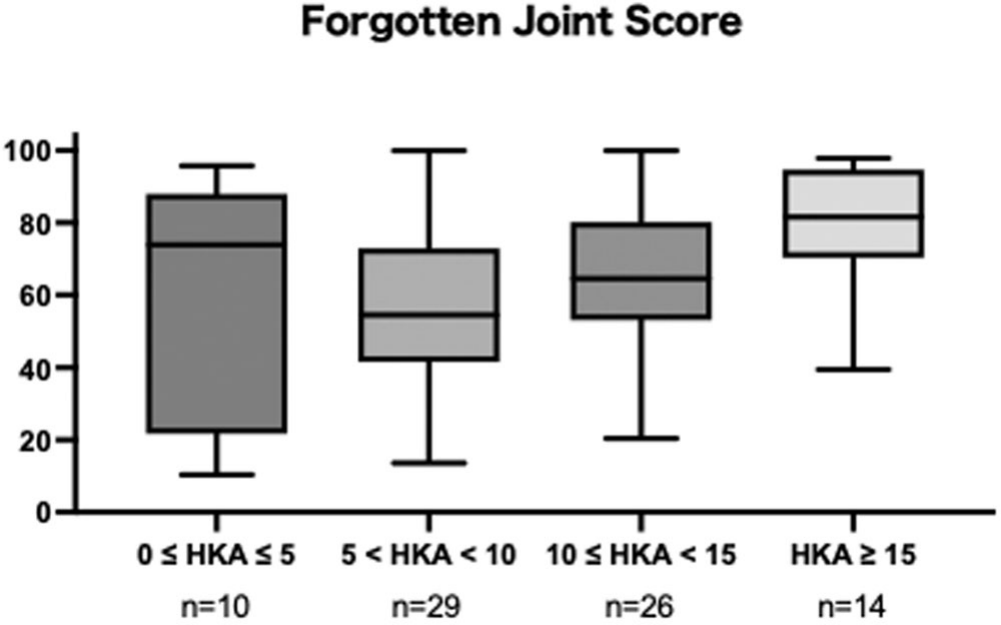
Forgotten Joint Score (FJS) according to pre‐operative coronal alignment. Box‐and‐whisker plots showing the FJS at 5 years after unrestricted kinematic alignment total knee arthroplasty, stratified by pre‐operative hip–knee–ankle angle (HKA): 0° ≤ HKA ≤ 5° (*n* = 10), 5° < HKA < 10° (*n* = 29), 10° ≤ HKA < 15° (*n* = 26) and HKA ≥ 15° (*n* = 14).

The distribution of FJS according to pre‐operative alignment is illustrated in Figure 1. Mean FJS values in the four alignment groups were 59 ± 34, 60 ± 24, 64 ± 24 and 80 ± 17, respectively. Although knees with severe pre‐operative varus deformity (HKA ≥ 15°) demonstrated numerically higher FJS values compared with the other alignment groups, this difference did not reach statistical significance (Kruskal–Wallis test, *p* = 0.078).

### Five‐year radiographic outcomes according to pre‐operative coronal alignment

Five‐year radiographic outcomes according to pre‐operative coronal alignment are shown in Table 4.

**Table 4:** Five‐year radiographic outcomes according to pre‐operative HKA group.

Outcome	0 ≤ HKA ≤ 5 (*n* = 25)	5 < HKA < 10 (*n* = 56)	10 ≤ HKA < 15 (*n* = 59)	HKA ≥ 15 (*n* = 44)	*p*‐Value
Post‐operative HKA, °	0 ± 3	2 ± 3	3 ± 4	6 ± 3	<0.0001
Change in HKA (post‐pre), °	3 ± 3	6 ± 3	9 ± 4	12 ± 3	<0.0001
Post‐operative mLDFA, °	86 ± 3	87 ± 2	89 ± 3	90 ± 3	<0.0001
Post‐operative MPTA, °	86 ± 2	85 ± 2	85 ± 3	84 ± 2	0.004

Abbreviations: HKA, hip–knee–ankle angle; mLDFA, mechanical lateral distal femoral angle; MPTA, medial proximal tibial angle.

Post‐operative HKA differed significantly among the four groups (*p* < 0.0001), with greater residual varus observed in knees with more severe pre‐operative deformity. Similarly, the magnitude of coronal correction, expressed as the change in HKA from pre‐operative to post‐operative measurements, increased progressively with increasing severity of pre‐operative varus alignment (*p* < 0.0001).

No revisions for any reason, including infection, periprosthetic fracture, or aseptic loosening, during the 5‐year follow‐up period.

## DISCUSSION

The most important finding of the present study was that urKA‐TKA provided excellent 5‐year clinical outcomes and implant survivorship in a Japanese population with a high prevalence of varus knee morphology. No revisions were required during the follow‐up period, and patient‐reported outcome measures demonstrated favourable mid‐term results. Importantly, even knees with severe pre‐operative varus deformity (HKA ≥ 15°) achieved comparable clinical outcomes to those with milder deformity, without evidence of compromised function or increased failure risk.

These findings support the concept that restoring each patient's native joint line orientation and soft‐tissue envelope does not compromise mid‐term outcomes, even in knees with pronounced coronal deformity [12, 19]. In contrast to MA strategies that aim to normalise limb alignment, urKA allows residual varus when it reflects the patient's constitutional morphology. This consideration may be particularly relevant in Japanese patients, in whom varus alignment patterns are more prevalent than those reported in Western populations [24, 27].

Several previous studies have reported favourable mid‐ to long‐term survivorship following KA‐TKA. In addition, early implant‐related failures after urKA‐TKA appear to be uncommon, as reported in a previous institutional analysis focusing on early failure patterns in Japanese patients [25]. Institutional series have demonstrated revision‐free survival rates exceeding 95% at more than 10 years, including reports with follow‐up extending to 16 years [6, 11, 28]. In addition, large‐scale registry analyses from Australia and New Zealand have demonstrated no increased risk of revision following KA‐TKA compared with MA‐TKA [15]. The present study extends these observations by confirming excellent 5‐year survivorship in an East Asian cohort treated exclusively with urKA principles, with no revisions observed during the study period.

The 5‐year PROMs observed in the present study are comparable to those reported in previous studies of KA‐TKA. Mid‐term KA‐TKA series have reported KSS satisfaction scores of approximately 25–30, KSS functional activity scores around 70–80 and FJS in the range of 60–75, indicating that the present results are consistent with established KA‐TKA outcomes [2, 6, 13, 31].

The present results are particularly relevant in the context of severe varus deformity, which has traditionally been considered a challenging condition in TKA. MA‐TKA in such knees often requires extensive soft‐tissue releases or compromises in component positioning to achieve a neutral axis, potentially altering native knee kinematics [20]. In contrast, urKA‐TKA aims to preserve the pre‐arthritic joint line and ligament balance, thereby avoiding excessive releases. In the current study, knees with severe varus deformity demonstrated clinical outcomes comparable to those with non‐severe varus alignment. Although FJS values were numerically higher in the severe varus group, this difference did not reach statistical significance when analysed across four alignment groups. This finding may reflect improved kinematic behaviour and a more physiologic feel of the knee when constitutional alignment is respected. Because the FJS is designed to capture subtle differences in joint awareness and is less affected by ceiling effects than conventional PROMs [1], this trend may indicate that preservation of patient‐specific coronal morphology allows the reconstructed knee to function within a chronically adapted soft‐tissue envelope. However, this finding should be interpreted cautiously given the limited sample size.

Umbrella reviews, which synthesise evidence across multiple systematic reviews and meta‐analyses, have recently highlighted the limitations of grouping heterogeneous KA techniques and emphasised the importance of precise radiographic reporting and consideration of patient‐specific morphology [4, 5, 9, 21]. In particular, recent studies have demonstrated that urKA does not compromise outcomes in varus knees and may even outperform MA in selected phenotypes, while yielding comparable results in others [8]. These findings suggest that varus severity alone is an insufficient criterion for rKA and that knee phenotype and native morphology should be considered when selecting alignment strategies.

Radiographic evaluation further supported the safety of urKA in this cohort. Only one knee demonstrated a radiolucent line at the tibial component, which was non‐progressive and did not lead to clinical symptoms or revision. No additional radiolucent lines were observed during follow‐up. These findings are consistent with previous reports indicating that residual varus alignment does not necessarily predispose to early loosening when the joint line orientation and load distribution are restored in a physiologic manner [17, 23]. These findings further underscore the limitations of a uniform alignment target and support alignment strategies that accommodate individual native coronal morphology rather than enforcing neutral MA [16, 26]. Regarding post‐operative valgus HKA observed in a subset of knees, this should be interpreted in the context of how pre‐operative HKA is measured. Pre‐operative HKA includes the influence of the joint line convergence angle (JLCA), which reflects intra‐articular deformity and soft‐tissue laxity. As a result, some knees with valgus bony morphology may still present with slight varus HKA pre‐operatively. After arthroplasty, restoration of joint line orientation and soft‐tissue balance can reduce the JLCA component, and these knees may therefore shift to a valgus HKA post‐operatively without representing technical error or overcorrection.

Similarly, pre‐operative MPTA measured in osteoarthritic knees may be underestimated due to medial bone loss and joint surface wear. After arthroplasty, restoration of joint surface geometry and soft‐tissue balance can result in post‐operative MPTA values that are closer to the native bony alignment. Therefore, a shift toward more neutral post‐operative MPTA in some knees with marked pre‐operative varus should not be interpreted as overcorrection, but rather as recovery toward the underlying constitutional alignment.

Several limitations of this study should be acknowledged. First, the retrospective design and incomplete 5‐year follow‐up introduce the potential for selection bias. However, no revisions or implant‐related complications were identified among patients prior to loss to follow‐up. Second, the lack of a MA control group precludes direct comparison between alignment strategies. Third, the cohort was treated using a single medial pivot implant design, which may limit generalisability. Fourth, although outcomes were analysed according to four pre‐operative coronal alignment groups, the sample size within each subgroup—particularly the severe varus group—was relatively small, which may have limited the statistical power to detect subtle differences in patient‐reported outcome measures. Finally, the number of knees with severe varus deformity was relatively small, and findings related to this subgroup should be interpreted with caution.

Despite these limitations, this study has several strengths. It represents one of the largest series reporting 5‐year outcomes of urKA‐TKA in Japanese patients, includes detailed radiographic analysis, and specifically examines outcomes in knees with severe varus deformity. The consistency of surgical technique and implant choice further strengthens the validity of the findings. These findings support the clinical applicability of urKA‐TKA in Japanese patients across a wide range of varus morphologies.

Further studies with longer follow‐up are warranted to confirm the durability of urKA, particularly in more complex cases and knees with severe deformity. In addition, future investigations focusing on cases in which post‐operative alignment diverges substantially from pre‐operative planning may provide further insights into factors influencing clinical variability.

## CONCLUSIONS

UrKA‐TKA provided excellent 5‐year clinical outcomes in a Japanese population with predominantly varus knee morphology, with no revisions observed during the 5‐year follow‐up period. Severe pre‐operative varus deformity did not adversely affect mid‐term outcomes, supporting the safe application of urKA‐TKA across a wide spectrum of varus alignment.

## AUTHOR CONTRIBUTIONS

Conceptualisation, methodology, validation, formal analysis, data curation, writing—original draft, and writing—review and editing: S.T. Investigation and resources: S.T., S.F., T.Y., I.T., and D.S. Supervision: S.F., T.N., and Y.W. All authors contributed to the writing of the final manuscript and approved the submitted version.

## CONFLICT OF INTEREST STATEMENT

Shuji Toyono, Shigenobu Fukushima, and Takao Yamamoto have received speaker honoraria from Medacta for educational activities related to kinematic alignment total knee arthroplasty. The remaining authors declare no conflicts of interest.

## ETHICS STATEMENT

This study was approved by the institutional review board of Saiseikai Yamagata Saisei Hospital (approval number: 2021‐477; November 17, 2021). Written informed consent was obtained from all patients.

## Data Availability

The data sets generated and/or analysed during the current study are not publicly available due to patient privacy and institutional restrictions but are available from the corresponding author on reasonable request and with approval from the institutional review board.
